# Analysis of tumour oxygenation in model animals on a phosphorescence lifetime based macro-imager

**DOI:** 10.1038/s41598-023-46224-7

**Published:** 2023-10-31

**Authors:** Alexander V. Zhdanov, Rajannya Sen, Ciaran Devoy, Liang Li, Mark Tangney, Dmitri B. Papkovsky

**Affiliations:** 1 School of Biochemistry and Cell Biology, University College Cork, Pharmacy Building, College Road, Cork, Ireland; 2 Cancer Research @UCC, University College Cork, Cork, Ireland

**Keywords:** Cancer, Biomarkers, Optics and photonics

## Abstract

Monitoring of tissue O_2_ is essential for cancer development and treatment, as hypoxic tumour regions develop resistance to radio- and chemotherapy. We describe a minimally invasive technique for the monitoring of tissue oxygenation in developing grafted tumours, which uses the new phosphorescence lifetime based Tpx3Cam imager. CT26 cells stained with a near-infrared emitting nanoparticulate O_2_ probe NanO2-IR were injected into mice to produce grafted tumours with characteristic phosphorescence. The tumours were allowed to develop for 3, 7, 10 and 17 days, with O_2_ imaging experiments performed on live and euthanised animals at different time points. Despite a marked trend towards decreased O_2_ in dead animals, their tumour areas produced phosphorescence lifetime values between 44 and 47 µs, which corresponded to hypoxic tissue with 5–20 μM O_2_. After the O_2_ imaging in animals, confocal Phosphorescence Lifetime Imaging Microscopy was conducted to examine the distribution of NanO2-IR probe in the tumours, which were excised, fixed and sliced for the purpose. The probe remained visible as bright and discrete ‘islands’ embedded in the tumour tissue until day 17 of tumour growth. Overall, this O_2_ macro-imaging method using NanO2-IR holds promise for long-term studies with grafted tumours in live animal models, providing quantitative 2D mapping of tissue O_2_.

## Introduction

Back in 1909, a markedly reduced response to the radiation of the skin with limited blood supply highlighted the significance of tissue oxygenation for radiotherapy^1^. The studies conducted by Gray in 1950s have shown that hypoxia causes resistance to radiation in various tissue models, including human lung cancers^2,3^. Mechanistically, upon irradiation of deoxygenated tissue, low levels of Reactive Oxygen Species (ROS) are produced, such that ROS-induced damage of DNA, proteins and lipids under hypoxia becomes much lower than at physiological normoxia. Thus, being an intrinsic feature of many tumours^4–7^, hypoxia has emerged as an attractive target for cancer therapy^8^.

Although tumours are very different by their origin and metabolism, the underlying mechanism of deep deoxygenation is common: rapid tumour growth with uncontrolled cell proliferation, increases consumption of oxygen (O_2_) and reduces its supply via blood flow to the ‘core’ regions of the tumour. The growing demand for O_2_ is partially compensated by increased angiogenesis in tumour tissues (e.g. due to activation of HIF-dependent signalling^9^), however O_2_ levels the tumours still remain low and heterogenous. The reported median O_2_ levels in human tumours vary from 0.3 to 0.4% in pancreatic and prostate cancers to 1.9–2.2% in lung cancer^4^. Core tumour areas with critically low pO_2_ start to fail their normal physiological functions and become necrotic^10^. Necrotisation, in turn, leads to subsequent inflammatory response and poor prognosis^11,12^.

Over the years various techniques have been applied to study tumour hypoxia^6,13–15^, however reliable quantitative data on O_2_ levels in various human and mammalian tumours (reviewed in McKeown^4^) were mostly produced on microelectrode based Eppendorf pO2 histography system. For many years, this invasive technique has been considered as ‘gold standard’ for tissue O_2_ measurement, although it requires special expertise to achieve correct insertion of the electrode in tumour regions^16–18^. Still, the electrode disrupts the tissues and provides only point measurements rather than whole distribution of O_2_ in the tumour. The alternative non-invasive approaches include Positron emission tomography (PET), Near-infrared spectroscopy (NIRS), Photoacoustic spectroscopy, Magnetic resonance imaging (MRI), Electron paramagnetic resonance spectroscopy (EPR) and Phosphorescence Lifetime Imaging (PLIM).

PET is an in vivo tomographic imaging technique where organic molecular markers labelled with positron-emitting radioisotopes are used to measure the extent of hypoxia in tumours. The tracers are usually injected intravenously or inhaled. As soon as the tracer reaches the target, PET scanner with an array of photoelectric crystals is used to quantify the hypoxia. However, PET has low anatomical resolution (~ 4–5 mm)^19–22^.

NIRS is an optical technique that continuously monitors regional tissue oxygenation by measuring haemoglobin saturation with the transmission and absorption of light. The technique uses light in near infrared region to perform real time measurements. The drawback of these systems is that they measure bound O_2_ in vasculature, rather than free O_2_ and tissue pO_2_^23–26^.

Photoacoustic imaging is a non-invasive hybrid imaging modality which allows optical contrast sensing with acoustic resolution in deep tissue^27,28^. In this method, photon energy from a short laser pulse is absorbed by the sample and converted into heat, leading to a thermal-elastic expansion. This induces a rise of local pressure and emission of acoustic waves. Photoacoustic imaging detects these acoustic signals generated by optical absorption from endogenous chromophores (deoxy-Hb, or exogenous contrast agents such as, nanoparticles and organic dyes. Although the method generates high resolution images, it is limited by optical attenuation^29^ and inaccuracies at depth beyond 1 mm^30^.

MRI can image tumour pO_2_ in vivo*,* usually upon intravenous injection of perfluorocarbon or fluorinated nitroimidazole probes. This method, referred as fluorine-19 (^19^F) oximetry, can report on absolute values of O_2_ concentration^31–33^. Blood oxygen level-dependent MRI (BOLD-MRI) measures fluctuations in the amount of oxygen bound to haemoglobin in the blood^34–36^, though it does not provide absolute O_2_ concentrations. Dynamic contrast-enhanced MRI (DCE-MRI) is another promising method that can measure blood flow and blood volume in tissues^37–39^.

EPR spectroscopy also uses exogenous probes to obtain information about tissue oxygenation^40–42^. Radical probes with unpaired electrons are sensitive to tissue environment, including O_2_. Changes in the EPR signal due to the interaction of the probe with O_2_ are used to determine pO_2_. However, the paramagnetic markers used show significant toxicity which limits their use in humans. Development of low toxicity probes and dedicated instrumentation for clinical applications will increase the utility of this technique.

Phosphorescence Lifetime Imaging Microscopy (PLIM) of O_2_ is a minimally invasive technique. It involves injection of a suitable phosphorescent probe into the vasculature or in tissue areas, which can then provide quantitative measurement of tissue pO_2_^43–45^. Several PLIM setups and phosphorescent probes have been described for mapping O_2_ in animal models. Thus, dendrimeric structures were chemically attached to the emitter, to encapsulate and protect it from interaction with sample components, and control the rate of oxygen quenching. Oxyphors R2 and G2 belong to this group^46,47^, so as PtP-C343, the two-photon excitable phosphorescent nanoprobe^48^, and the latest enhanced two-photon excitable Oxyphor 2P probe which provides high sensitivity and O_2_ imaging at depths up to 600 µm^49^. However, these probes are rather complex and usually administered intravenously in live animals or by pressure injection into tissues. These probes cannot effectively penetrate cells and biological membranes and do not allow long-term use (only several hours).

NanO2-IR is another type of O_2_ sensing probe which consists of polymeric nanoparticles impregnated with a near-infrared phosphorescent dyes. NanO2-IR is cell-permeable, it can stain different cells and tissues and be applied topically^50,51^. Potentially, it can provide quantitative visualisation of O_2_ in long-term imaging experiments with animal models.

Recently we have described an imager based on the Tpx3Cam chip and TCSPC hardware, which was optimised for macroscopic PLIM imaging and biological applications^52^. The imager can detect individual photons and record their arrival time and time over threshold at each pixel simultaneously with good spatial and temporal resolution. This imager, in conjunction with the NanO2-IR probe, was successfully applied to imaging of O_2_ concentration in tissue samples ex-vivo^53–56^. In this study, we aim to demonstrate the Tpx3Cam imager in in vivo experiments—to map O2 distribution and tissue hypoxia in grafted tumours of laboratory animals (mice).

## Materials and methods

The cell-permeable nanoparticle probe NanO2-IR was synthesised as described previously^57,50^. Dulbecco's modified Eagle medium (DMEM), foetal bovine serum (FBS), paraformaldehyde (PFA), low melting point agarose, and all other chemicals were from Millipore-Sigma (Burlington, MA, USA).

### Cell culture and staining with NanO2-IR

Murine colorectal carcinoma CT26 cells (the American Collections of Cell Cultures) were maintained in high glucose DMEM supplemented with 10% FBS, 2 mM L-glutamine, 100 U/ml penicillin/100 μg/ml streptomycin, and 10 mM HEPES, pH 7.2 (complete DMEM), in humidified atmosphere of 95% air and 5% CO_2_ at 37 °C.

For staining, cells were seeded in wells of a 6-well plate (Sarstedt, Ireland) at 8 × 10^5^ cells per well and allowed to grow for 24 h in complete DMEM, then washed with serum-free DMEM, and incubated for 18–20 h with different concentrations of NanO2-IR in serum-free DMEM. Cells were then washed sequentially with serum-free DMEM, complete DMEM and PBS, trypsinised, transferred into serum-free DMEM and prepared for injection in animals.

As a viability reporter, total ATP levels in CT26 cells were analysed with CellTiter-Glo assay kit (Promega), according to the manufacturers’ protocol. Briefly, cells were incubated with different concentrations of NanO2-IR in FBS-free DMEM (100 μl, 18 h in a CO_2_ incubator), then the cells were lysed with 100 μl of CellTiterGlo reagent. After intensive shaking (2 min, 37 °C), the samples (200 μl) were incubated for 4 min, transferred into wells of white 96-well plates (Greiner Bio One) and analysed on a Victor 2 plate reader under standard luminescence settings.

### Experiments with animals (mice)

All animal procedures were performed according to the European Union Directive 2010/63/EU amended by Regulation (EU)2019/1010, as well as national ethical guidelines of the Health Products Regulatory Authority (HPRA), under project authorisation AE19130/I285. Health status of all mice was monitored daily for the duration of experiments. There were no deaths outside the humane euthanasia. Mice were humanely euthanized by cervical dislocation after tumours reached the size of 1.5 × 1.5 cm in diameter.

### Probe delivery and tumour induction in mice

Mice were kept at constant room temperature (22 °C) with natural day/night light cycle in a conventional animal unit. Standard laboratory food and water were provided ad libitum. Before experiments, the mice were given an adaptation period of at least 7 days. Female Balb/C (CT26 models) mice in good condition, disease free, weighted 16–22 g at 6–8 weeks of age (Envigo, U.K.), were included in the experiments. For routine tumour induction, a minimum tumorigenic dose of cells (1 × 10^5^ of non-stained CT26 cells or a mixture of 1 × 10^5^ non-stained cells and 1 × 10^5^ stained with NanO2-IR cells (unless otherwise stated) suspended in 200 μl serum-free culture medium were injected subcutaneously (SC) into the right flank. Following tumour establishment, tumours were allowed to grow and develop, and they were monitored daily. Tumour volume was measured and calculated according to the formula: V = (L × W^2^)/2, where L is the longest diameter of the tumour (length) and W is the longest diameter perpendicular to diameter L (width).

### Imaging tissue O_2_ on the Tpx3Cam imager

The PLIM macro-imager (described in Sen et al.^52^) comprised a Tpx3Cam optical camera (Amsterdam Scientific Instruments, The Netherlands), coupled by means of a Cricket adapter (Photonis, France) with an image intensifier (Photonis, France), a 760 ± 50 nm bandpass emission filter (25 mm, Edmund Optics, York, UK) and a 50 mm lens Navitar NMV-50M11 (Thorlabs). A bright 5 mm red LED (627 nm, Parts Express, Springboro, OH) was used for pulsed excitation of grafted tumours in animals. The Cricket adaptor provided a compact and flexible design to the imager which allowed 360° rotation. In this study the imager was used in an upright orientation, and it provided spatial resolution of 39.4 µm in X–Y plane and averaged signals in Z-dimension with maximal depth of light penetration into tissue of ~ 0.5 mm^52^.

On the day of imaging at defined time-points, mice were taken from the animal facility for biological and imaging experiments. One animal was taken, immobilised by concussion and tissue O_2_ was immediately imaged in vivo. The mouse was then euthanized by cervical dislocation and immediately subjected to O_2_ analysis in live post-mortem tissue, taking a series of five PLIM imaging sessions with 6 min intervals. During the imaging, the animal was kept in a petri dish on a temperature-controlled stage with a block heater (Thermo Fisher Scientific, Waltham, MA) set at 37 °C, such that tumour area was facing the camera. This procedure was repeated for each animal in the group. Body temperature of the mouse at different time points of the imaging procedure was traced using rectal T-probe Ellab DM852 (Medical Precision) and IR DT8803 (Duratool) thermometer.

PLIM images were acquired at LED power 2.4 V, pulse width 500 ns and integration time 20 s. Other operational parameters and imaging focus were adjusted with Sophy software provided by Amsterdam Scientific Instruments. The raw data was acquired by a custom designed software and then post-processed with a C-language program code. *Tri2* software was used to fit the data with two-exponential decay functions and determine the lifetime values.

### Imaging on a confocal PLIM microscope

For the analysis of NanO2-IR distribution in tissue, tumours were excised immediately after the post-mortem animal imaging and fixed in 4% paraformaldehyde in PBS (30 min at room temperature). The samples were then washed in PBS, embedded into low melting point agarose (4% in PBS) and sectioned on a Leica VT1000s vibratome (Leica Biosystems, Wetzlar, Germany) to produce ~ 500 μm thick slices of the middle part of tumour cross-section, which were imaged. Prior to confocal imaging, NanO2-IR isles inside the slices were visualised with a camera of iPhone 14 (Apple, CA).

Microscopy of the NanO2-IR probe was performed on the confocal FLIM/PLIM imaging system (Becker&Hickl, Germany): at 37 °C for live cells using a 20 ×/1.0 W-Plan Apochromat dipping objective, and at 22 °C for tissue slices using a 5 ×/0.25 FLUAR objective. Phosphorescence lifetime signals were measured in air-saturated conditions (for the cells and tumour tissues) and after chemical deoxygenation by the addition of 5 mg/ml Na_2_SO_3_ and 5 mg/ml K_2_HPO_4_ (tumour tissues). The imaging system comprised an upright microscope Axio Examiner Z1 with heated incubator and stage (T = 37 °C), motorized Z-axis control (all from Carl Zeiss, Germany)^58^, connected to a DCS-120 confocal scanner (B&H), a 405 nm BDL-405-SMNi picosecond diode laser (B&H), and an R10467U-40 photon counting detector, with > 30% quantum efficiency at 400 ± 700 nm (Hamamatsu Photonics, Japan). Excitation and emission light was separated with a 405 nm dichroic mirror and a 716 ± 20 nm emission filter (both from Semrock, Rochester, NY, USA). The laser and scanner were connected to the TCSPC detection hardware (B&H), with scanning resolution 256 × 256 pixel. Image acquisition was controlled by MicroToolBox software, version 2011 (Carl Zeiss) and SPCM software, version 9.81 (B&H). The PLIM data was then processed in SPCImage software, version 8.4 (B&H) to determine lifetime values. Numerical values corresponding to PLIM colour images and lifetime distribution histograms were exported in Microsoft Excel. Figures were assembled using ImageJ, Adobe Photoshop and Illustrator software.

### Ethical approval

All animal procedures were performed under authorisations issued by the Health Products Regulatory Authority (HPRA, Ireland), under project authorisation AE19130/I285, in accordance with the European Communities Council Directive (2010/63/EU) and amended by Regulation (EU)2019/1010. All experimental protocols were approved by the Animal Experimentation Ethics Committee of the University College Cork. The reporting in the manuscript follows the recommendations in the ARRIVE guidelines.

## Results and discussion

### Optimisation of NanO2-IR probe delivery and tumour growth

First, we optimised conditions for delivery of NanO2-IR probe in mice and its incorporation in tumours formed by CT26 cells injected subcutaneously. For that, we examined cell staining and toxicity effects of the probe on cultured cells, and optimised conditions for producing grafted tumours stained with NanO2-IR. This work allowed us to choose staining concentrations of NanO2-IR for cell staining that produce measurable phosphorescence during tumour growth, which was monitored for up to 17 days.

**Figure 1:**
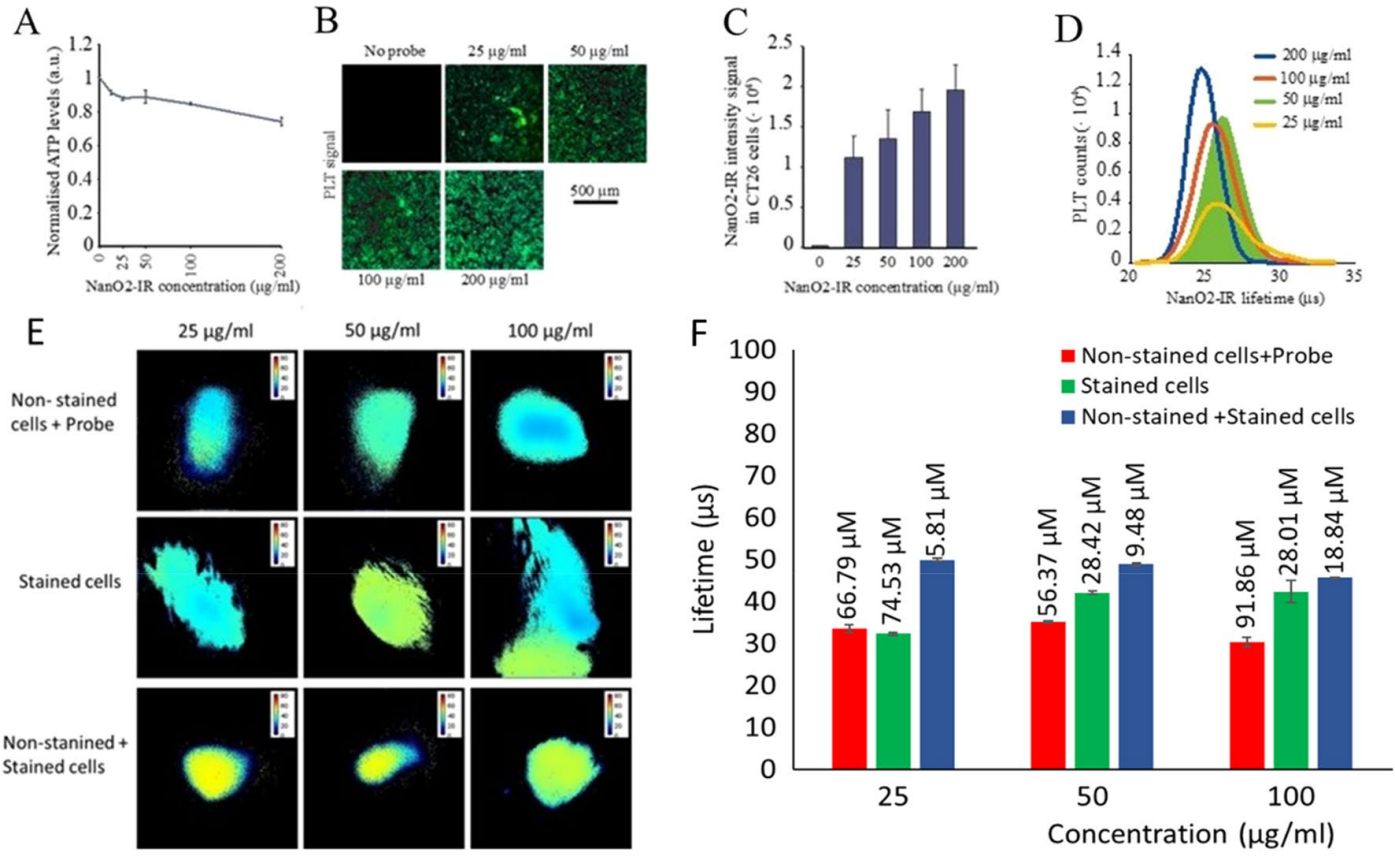
Optimisation of NanO2-IR delivery to grafted tumours in mice. (**A**) Effects of the different concentrations of NanO2-IR probe (indicated, incubation time 18 h, N = 6) on the viability of cultured CT26 cells. Viability was analysed with a CellTiter-Glo kit, which measures total cellular ATP levels. (**B**, **C**) Confocal microscopy analysis of staining efficiency of CT26 cell at different concentrations of NanO2-IR (18 h, N = 6). (**D**) Distribution histogram of lifetime values, calculated for the cells stained with different concentrations of NanO2-IR (as in **B**, **C**). (**E**) Post-mortem PLIM images of NanO2-IR in tumour tissue, taken with Tpx3Cam imager; the probe was delivered as specified. (**F**) Bar charts comparing probe lifetime values and calculated O_2_ concentrations for the different trials (as in **E**, N = 3).

The CellTiterGlo cytotoxicity assays did not show major effects on cells of the NanO2-IR probe used at concentrations 0–200 µg/ml and incubation time 18 h (Fig. 1A). However, microscopy analysis revealed some long-term effects of NanO2-IR: the cells treated with > 50 µg/ml probe for 20 h did not proliferate and died within 20–30 h after the staining (Fig. S1A). Even at 50 µg/ml, the probe strongly reduced cell proliferation rate, which gradually increased at lower NanO2-IR concentrations.

These in vitro results suggested that development of tumours from cells pre-stained with NanO2-IR at > 50 µg/ml, or even lower concentrations, could be problematic. Therefore, we tested proliferation of 1:1 mixtures of non-stained and stained cells with different concentrations of NanO2-IR. This strategy markedly improved cell proliferation in vivo: even in the presence of cells stained with 200 µg/ml NanO2-IR, the non-stained cells grew well and formed tumours (Fig. S1B). So, injection of a mixture of stained and non-stained cells produced efficient tumour growth and sufficient quantities of the probe in tumour tissue. As an alternative, we also administered non-stained cells mixed with NanO2-IR just before the injection.

Next, we examined cell staining efficiency at different NanO2-IR concentrations. When analysing phosphorescence of NanO2-IR in the stained CT26 cells on a confocal PLIM microscope, we noticed that intensity signals do not increase proportionally with probe concentration used for the staining. Thus, when NanO2-IR levels in the medium increased from 25 µg/ml to 200 µg/ml, signals produced by cells increased by less than 50% (Fig. 1B,C).

When used at concentrations 25–200 µg/ml, NanO2-IR produced high and sustained intensity signals in cells and stable lifetime values, which were usable for the analysis of tissue oxygenation in mice on the PLIM macro-imager (Fig. 1D). To prove this, we injected the cells with the probe in the right flank areas of freshly sacrificed animals, and then conducted imaging of these areas. NanO2-IR probe was delivered into animal tissue at three different concentrations (25, 50, 100 µg/ml) using three different methods: (1) non-stained cells mixed with the probe; (2) cells stained with probe; and (3) 1:1 mixture of the non-stained and stained cells. For the first two methods, the injections were made to post-mortem animals and images were taken within 20 min. For the third method, (called here Preliminary Trial), the injections were made in live animals, and the images were taken on the days 4, 7 and 10 of tumour growth—from 3 mice on each Day. For each animal, the first measurement was taken from live anaesthetized animal (time zero), and then five repeated measurements over 6–30 min were taken from euthanized animal. Thus, the total number of PLIM images in this Trial, N = 54 (3 × 6 × 3).

Figure 1E shows PLIM images for the three methods of delivery at three different concentrations of NanO2-IR probe, taken immediately after the animals were euthanised, on Day 4 of tumour growth. Figure 1F demonstrates the corresponding lifetime values and O_2_ concentrations, calculated using the calibration curve and transformation equation generated for the NanO2-IR probe in solution at 30 °C (Figure S4, Supplementary Information). From these experiments, it appears that the mixture of non-stained and cells stained with all three probe concentrations produced the most stable lifetime signals and lowest O_2_ values, which were expected in the tissue of euthanised animals.

**Figure 2:**
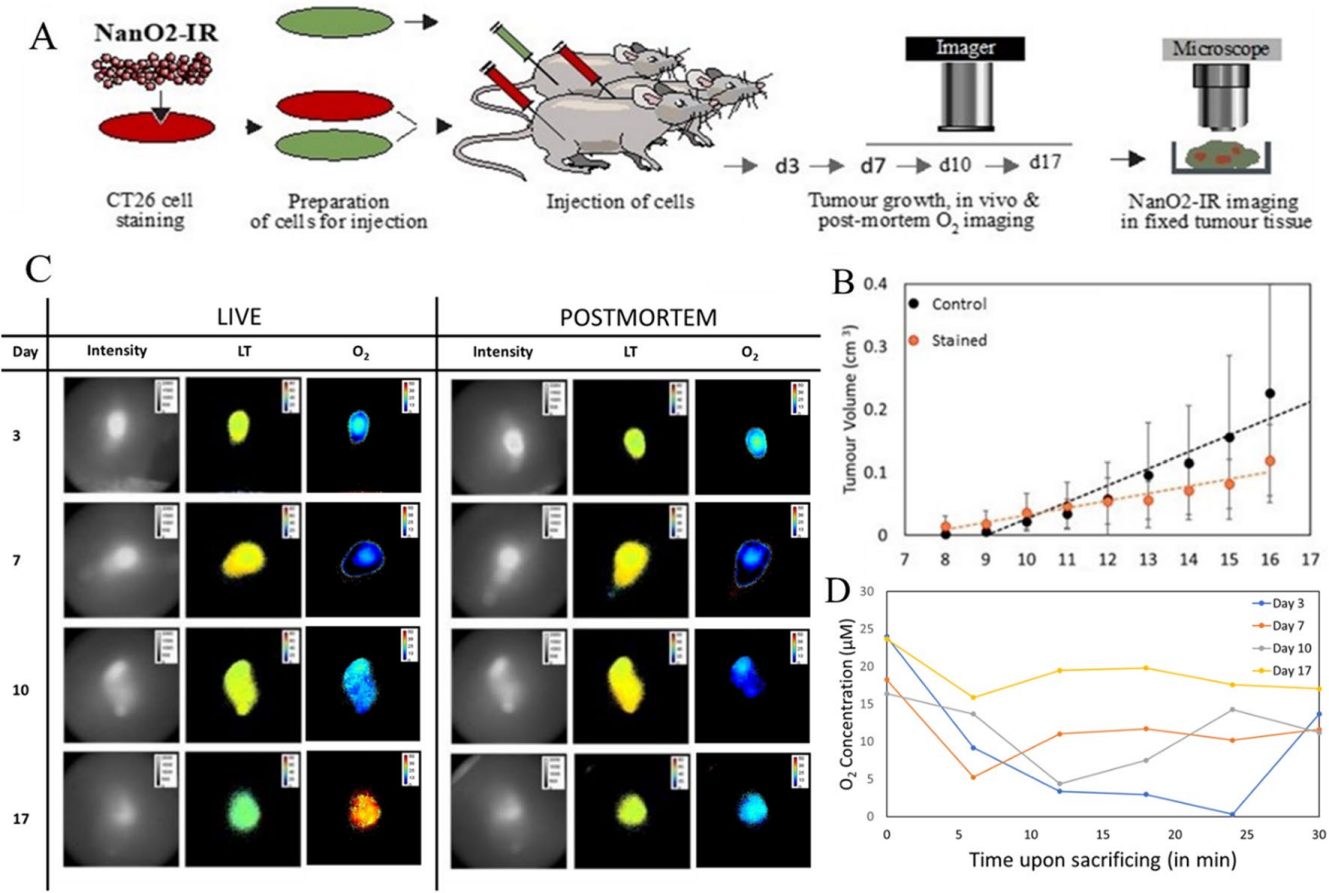
Imaging analysis of tumour oxygenation. (**A**) The scheme of in vivo and post-mortem O_2_ imaging of tumour tissue of mice. CT26 cells stained with NanO2-IR (50 μg/ml, 18 h) were mixed with non-stained CT26 cells (1:1) and injected immediately in mice subcutaneously (right flanks); tumours were allowed to grow for up to 17 days, and then were analysed on the Tpx3Cam PLIM imager on days 3, 7, 10 and 17 in vivo and immediately after animal’s death. After this NanO2-IR distribution was analysed in fixed tumour tissues on the confocal PLIM microscope. Animals in control group (tumour size and phosphorescence background controls) were injected with non-stained CT26 cells. N = 6 in all animal groups. (**B**) Comparison of the growth rate of the control and stained tumours. (**C**) Intensity and PLIM images of NanO2-IR in live and euthanised animals on different days of tumour growth. (**D**) The O_2_ concentration values of the probe in tumour regions of live and euthanised animals, taken with 6 min intervals on the different days of tumour growth (N = 3 in each group).

Based on the results of cell staining, analysis of proliferation and post-mortem animal imaging in the Preliminary Trial, for in vivo O_2_ imaging experiments of the Main Trial we have chosen the injection of 1:1 mixture of stained with 50 µg/ml probe and non-stained cells (Fig. 2A).

### In vivo and post-mortem analysis of O_2_ levels in tumours

We ruled out repeated imaging of live animals under anaesthesia, which is known to alter functioning of the respiratory system and oxygenation state of animal tissue^59^. Such a procedure would also require sterilizing the imager and placing it inside the animal house, which was not feasible. In our experimental procedure, the imager was set up next to the animal facility. For imaging, test animals were placed on a heated stage. As tested with the rectal and IR thermometers, animal and tumour temperature during the imaging sessions stayed relatively con616161stant at around 30–33 °C (Figure S5, Supplementary Information). This allowed us to apply to all post-mortem images the O_2_ calibration generated in vitro at 30 °C.

In the Main Trial, the end-point O_2_ imaging analysis of animals was performed on days 3, 7, 10 and 17 of tumour growth (Fig. 2A). Grafted tumours stained with NanO2-IR were seen to grow approximately two times slower than those produced with unstained CT26 cells (Fig. 2B). This is in line with the above toxic effects of the probe on cells. By day 17, tumour size reached ~ 0.12 cm^3^, as compared to ~ 0.23 cm^3^ for control group. Figure 2C shows that at all stages of growth, tumour regions were clearly detectable on the intensity images, and this allowed accurate calculation of phosphorescence lifetime values. On the different days of tumour growth, mean lifetime values for standard 30 min imaging sessions performed on the different Days ranged between 44 and 52 µs, which correspond to tissue O_2_ concentrations 25–0 μM, respectively (Fig. 2D). Similar [O_2_] profiles for individual animals (Mouse 1 from each group) are shown in Supplementary Figure S6.

**Figure 3:**
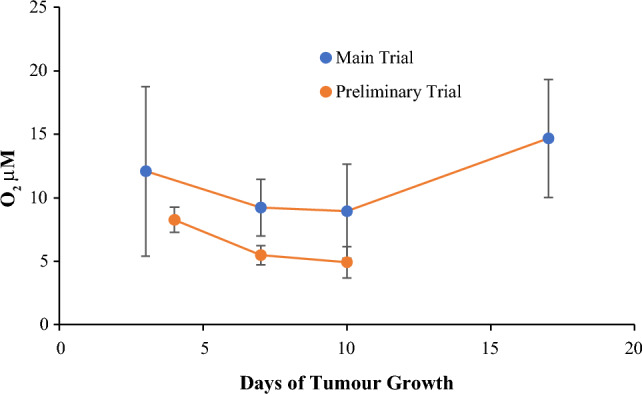
Dynamics of tumour oxygenation. Five PLIM images were taken at 6–30 min after animals were euthanised. Data of the Main and Preliminary Trials are presented. For each time point, N = 3 in both Trials.

The profile of mean [O_2_] over the whole period of tumour development and PLIM measurements under the Main Trial (Days 3, 7, 10 and 17; N = 3 animals for each time point, 6 measurements per animal) is shown in Fig. 3. Moreover, since the tumour growth and imaging conditions in the Preliminary Trial were identical to the Main Trial, the results of Preliminary Trial (i.e., mean [O_2_] on Days 4, 7 and 10; N = 3 animals for each time point, 6 measurements per animal) were also plotted on Fig. 3 and analysed as the second set of independent experiments. The two profiles show similar [O_2_] values, with a minor downward trend from Day 3 (or Day 4) to Day 10, and a prominent increase in tumour [O_2_] at the end of the Main Trial on Day 17. We attribute the latter effect to: (1) reduced O_2_ consumption by tumours having large necrotic areas, which develop and expand in subcutaneous tumours on Day 14 and thereafter^60^; and (2) more heterogenous [O_2_] distribution, as ‘a branched and chaotic vascular system’ in the tumour becomes dominant on Day 17 after the injection of CT26 cells^61^. Indeed, the large variability of [O_2_] values measured on Day 17 (Fig. 3) supports this hypothesis.

However, statistical analysis of the PLIM data (Fig. 2D) did not reveal significant differences between tumours grown for different periods of time, likely because of the variability between animals and intratumoural processes. Lifetime values for the live animals ranged from ~ 44 µs (days 3 and 17) to ~ 46 µs (days 10 and 14), and the repeated measures ANOVA test did not show significant difference in lifetime values between PLIM images of live and post-mortem tumour tissue taken during each individual day of tumour growth. At the same time, ANOVA test showed a trend towards increased LT values in the euthanised vs live animals (see statistical details in Fig. 2C). The trend was particularly strong for day 17, with *p* = 0.131 for live vs dead mice.

It is generally accepted that soon after death, when blood circulation stops, tissue O_2_ gets rapidly depleted. The observed little difference in NanO2-IR lifetime values between the live and euthanised animals on Days 7 and 10 of tumour progression, suggested that tissue oxygenation was rather low at these stages. And on Days 3 and 4 of the Main and Preliminary Trials, when tumours were not palpable, O_2_ showed a positive trend compared to Days 7 and 10, but were still lower than on Day 17 (Fig. 2B). Day 3 of the Main Trial showed the highest variability of tissue [O_2_] values, the reasons of which are not clear. Similar effects were reported for the other cell models^62,63^. So, active proliferation of the injected CT26 cells during the initial engraftment phase can be accompanied by increased fraction of ‘hypoxic cells inside the growing tumour’, detectable by the imager.

### Statistical analysis

The repeated measures ANOVA test was performed on the lifetime values recorded on the different days (shown in Fig. 2D), which shows:

- On day 3, for different imaging time F(1.05, 2.1) = 1.89, *p* = 0.301, with a mean of 43.7 µs for 0 min, 48.9 µs for 6 min, 50.59 µs for 12 min, 50.7 µs for 18 min, 51.38 µs for 24 min. F = 5.2, *p* = 0.3857 for 0 min vs 6 min; F = 6.889, *p* = 0.2672 for 0 min vs 12 min; F = 6.999, *p* = 0.3215 for 0 min vs 18 min; F = 7.684, *p* = 0.2839 for 0 min vs 24 min.
- On day 7, for different imaging time, F(1.45, 2.89) = 2.44, *p* = 0.232, with a mean of 45.8 µs for 0 min, 50.1 µs for 6 min, 48.3 µs for 12 min, 48.1 µs for 18 min, 48.6 µs for 24 min. F = 4.274, *p* = 0.2143 for 0 min vs 6 min; F = 2.515, *p* = 0.1808 for 0 min vs 12 min; F = 2.298, *p* = 0.165 for 0 min vs 18 min; F = 2.783, *p* = 0.2031 for 0 min vs 24 min.
- On day 10, for different imaging time F(4, 8) = 2.24, *p* = 0.154, with a mean of 46.5 µs for 0 min, 47.4 µs for 6 min, 50.3 µs for 12 min, 49.4 µs for 18 min, 47.2 µs for 24 min. F = 0.951, *p* = 0.2469 for 0 min vs 6 min; F = 3.8277, *p* = 0.144 for 0 min vs 12 min; F = 2.928, *p *= 0.2507 for 0 min vs 18 min; F = 0.7453, p = 0.5331 for 0 min vs 24 min.
- On day 17, for different imaging time, F(1.8, 3.59) = 3.79, *p* = 0.131, with a mean of 43.8 µs for 0 min, 46.7 µs for 6 min, 45.3 µs for 12 min, 45.2 µs for 18 min, 46.0 µs for 24 min. F = 2.85, *p* = 0.0746 for 0 min vs 6 min; F = 1.5387, *p* = 0.0214 for 0 min vs 12 min; F = 1.4177, *p *= 0.1182 for 0 min vs 18 min; F = 2.2347, *p* = 0.1981 for 0 min vs 24 min.

### Analysis of NanO2-IR distribution in tumour tissue

**Figure 4:**
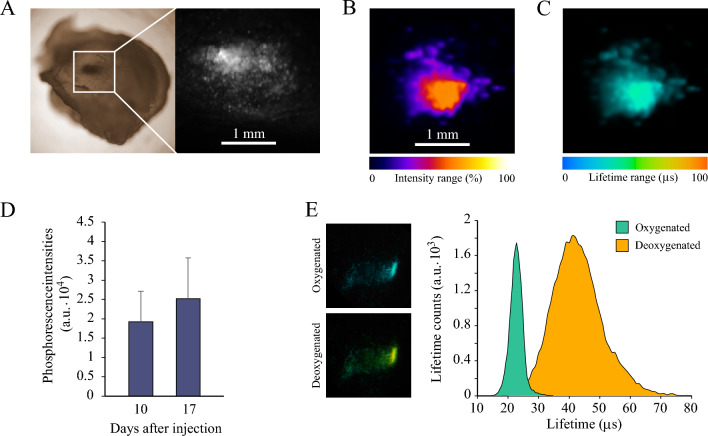
Analysis of NanO2-IR localisation in tumour tissue. (**A**) Representative image of tumour tissue with a NanO2-IR ‘isle’ positioned in the middle (left). The corresponding phosphorescence intensity image obtained by confocal PLIM microscopy (right, 5 × magnification). (**B**, **C**) Typical span of the phosphorescence intensity signals across the NanO2-IR ‘isle’ (B) and the corresponding lifetime values (C). (**D**) Analysis of the intensity signal of NanO2-IR residing in tumour tissue at different stages of tumour development (N = 6). (**E**) Response of NanO2-IR ‘isle’ to chemical deoxygenation of the tissue by sulphite. Right panel shows the typical shift of the distribution of lifetime values in response to decreased O_2_ levels. Images represent stacks of six (**A**–**C**) or three (**E**) focal planes taken in 5 µm step.

We also studied localisation of NanO2-IR in tumour tissue. For this, after the in vivo and post-mortem O_2_ imaging sessions with animals, tumours were excised, fixed, sliced on a vibratome and then examined on a confocal PLIM microscope for the presence and distribution of the probe. In most cases, tissue areas containing NanO2-IR probe were detectable by visual examination, as darker spots (Fig. 4A). Further analysis confirmed the presence of NanO2-IR in these ‘isles’, surrounded most by non-stained tissue or tissue with low levels of the probe distributed diffusely. Such patchy distribution of the probe suggested that stained CT26 cells died soon after the injection and their debris formed these ‘isles’ of NanO2-IR probe, which were 0.1–3 mm in size (mostly 0.2–1 mm). The ‘isles’ were surrounded by actively growing non-stained cells, which produced tumours similar in size to the control (non-stained) tumours.

Using 3D image reconstructions, we also analysed LT signals across the stained ‘isles’ and found that they were independent on the probe concentration and aggregation (Fig. 4B,C). NanO2-IR ‘isles’ also remained unaffected during tumour growth: their size and average intensity signals were similar in tumour samples collected on days 10 and 17 (Fig. 4D, Fig. S3). The median lifetime values in the tissues collected at different stages of tumour growth also did not change and stayed at 22–24 µs. Upon chemical deoxygenation, the lifetimes increased to > 40 µs. This confirms the specificity of the observed phosphorescent signals from the probe, which were not affected by sample preparation, including the fixation with paraformaldehyde (Fig. 4E).

## Conclusions

The Tpx3Cam PLIIM imager and NanO2-IR probe showed promising performance in fast quantitative imaging of tissue hypoxia in animal models with grafted tumours. The conditions of NanO2-IR use in animals were optimised to minimise adverse effects of the probe on grafted tumours and enable long-term retention of the probe in grafts. The imager produced detailed lifetime maps that reflected hypoxic conditions in tumour regions, both in live and euthanised animals. The lifetime values measured in live animals ranged within 44–46 µs from day 3 to day 17 of tumour growth and showed no correlation with tumour progression. These results were in line with previous studies demonstrating profound hypoxia in the tumour tissue. The distribution of the NanO2-IR probe in the tumours and its LT signal in oxygenated and deoxygenated conditions were also examined by confocal PLIM microscopy of tissue samples which were excised on days 3–17. Compared to the other O_2_ sensitive phosphorescent probes intended for intravital use, NanO2-IR was seen to be the most long-lasting probe, which produces stable and easily detectable intensity and lifetime signals for at least 17 days, little affected by tissue environment and tumour growth. Overall, the study demonstrated a fast, high-resolution, quantitative, minimally invasive method of PLIM imaging in physiological in-vivo studies.

### Supplementary Information

Supplementary Information.

## Data Availability

Data available on request from the corresponding author.
