# Supplementary material for: Analysis of tumour oxygenation in model animals on a phosphorescence lifetime based macro-imager
Source: Sci Rep. 2023 Oct 31;13:18732. doi: 10.1038/s41598-023-46224-7 (PMC10618169; doi:10.1038/s41598-023-46224-7)
Supplement: Supplementary file 1 — Supplementary Information. [file 41598_2023_46224_MOESM1_ESM.docx]

Supplemental Material

**Analysis of tumour oxygenation in model animals on a phosphorescence lifetime based macro-imager**

*Alexander V. Zhdanov, Rajannya Sen, Ciaran Devoy, Liang Li, Mark Tangney, Dmitri B. Papkovsky*

**Figure S1**. Effects of the NanO2-IR probe on CT26 cell proliferation capacity. **A**. Cells were stained for ~20 h with the probe at the indicated concentrations (top panel), washed, re-plated and grown for further 28 h (bottom panel). **B**. Cells stained as in (**A**) with 200 μg/ml probe were mixed with non-stained cells (1:1), re-plated and grown for 28 h (right panel); non-stained cells (left) and stained cells (middle) are shown as controls for maximal and minimal proliferation, respectively. Bars indicate ~100 μm scale. Images were taken using a light microscope and an iPhone camera.


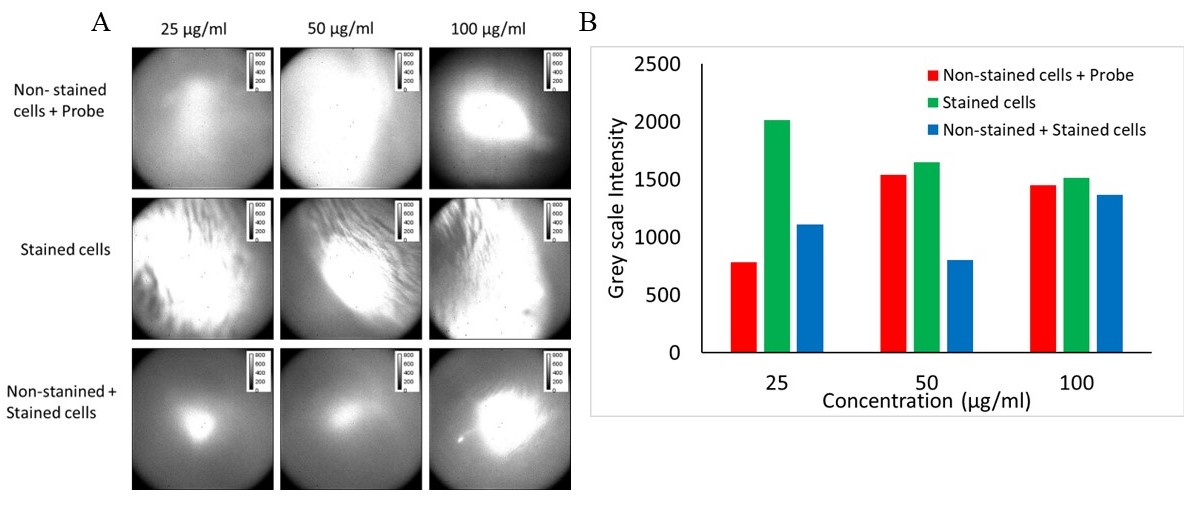


**Figure S2**. Optimisation of the NanO2-IR probe delivery to grafted tumours in mice. **A.** Grey-scale intensity images of tumour tissue with different means of NanO2-IR delivery. **F.** Bar charts showing the intensity signals for the different methods.

**Figure S3**. Phosphorescence intensity signals of the NanO2-IR probe in tumour tissue at different stages of tumour development. Images in (**A**) were collected using confocal PLIM microscope at 5x magnification. **B**. Quantitative analysis of NanO2-IR signals in tumour tissue on days 10, 14 and 17(N=16); tissue with no NanO2-IR was used as background control.

**Figure S4**. O_2_ calibration curve and transformation equation for the NanO_2_-IR probe, generated in aqueous solution, at 30 ^°^C using Optech (Mocon) sensor reader as described in ^1^.

**Figure S5**. Time profiles of body/tumour temperature during the imaging session, measured in sacrificed animals with a rectal temperature probe (blue curve) and an IR-thermometer. N=3 for each time point.

**Figure S6**. Representative time profiles of [O_2_], produced in one imaging session with individual animal (Mouse 1), on the different days of the Main Trial (specified).

**References:**

1. Kelly, C. A. *et al.* Extruded phosphorescence based oxygen sensors for large-scale packaging applications. *Sensors and Actuators B: Chemical* 127357 (2019). doi:10.1016/j.snb.2019.127357.
